# Prevalence of Pediatric Preventive Dental Visits Among Children in Saudi Arabia: A Cross-Sectional Study

**DOI:** 10.3390/healthcare13192413

**Published:** 2025-09-24

**Authors:** Mohammed H. Alshamrani, Waad E. Alsaadi, Reem A. Alajlan, Amjad M. Alabdulmohsen, Ghada Saeed Alqahtani, Mannaa K. Aldowsari

**Affiliations:** 1 Department of Pediatric Dentistry and Orthodontics, College of Dentistry, King Saud University, Riyadh 11545, Saudi Arabia; walsaadi@ksu.edu.sa (W.E.A.); ralajlan@ksu.edu.sa (R.A.A.); amjad@ksu.edu.sa (A.M.A.); maldowsari@ksu.edu.sa (M.K.A.); 2 College of Dentistry, King Saud University, Riyadh 11545, Saudi Arabia; gs.qhtani@gmail.com

**Keywords:** pediatric dentistry, first dental visit, preventive dental care, AAPD guidelines, Saudi Arabia, parental awareness, oral health, cross-sectional study, early childhood caries, dental compliance

## Abstract

Background: Early preventive dental visits are critical to reducing oral diseases in children and establishing lifelong oral hygiene behaviors. The American Academy of Pediatric Dentistry (AAPD) recommends that a child’s first dental visit occur by age one. However, in Saudi Arabia, limited evidence exists on parental awareness, attitudes, and barriers toward this recommendation. Objective: This study aimed to evaluate the timing of the first dental visit among Saudi children and to identify parental knowledge, barriers, and sociodemographic factors associated with compliance with AAPD guidelines. Materials and Methods: A cross-sectional survey was conducted between April and July 2025 at the Pediatric Dental Clinic, King Saud University, Riyadh. A validated, self-administered Arabic questionnaire was distributed both online and in-clinic to Saudi parents of children under 12 years. A total of 465 completed responses were analyzed using descriptive statistics, chi-square tests, logistic regression, and Spearman correlation to assess associations between parental awareness, socioeconomic variables, and compliance. Results: Of the 465 respondents, 39.6% were aware of AAPD guidelines, yet only 30.5% reported compliance with the recommendation of a dental visit by age one. The most cited barriers were lack of time (42%), difficulty accessing clinics (22%), shortage of pediatric dentists (20%), and lack of awareness (16%). Higher parental education (*p* = 0.003) and income (*p* < 0.001) were significantly associated with compliance. A moderate positive correlation was observed between early dental visits and regular annual check-ups (ρ = 0.319). Conclusions: Compliance with AAPD guidelines among Saudi parents remains low, largely due to limited awareness and access barriers. Strengthening parental education, community-based programs, and affordable pediatric dental services is essential to promote preventive care and improve oral health outcomes in children.

## 1. Introduction

Oral health is one of the important factors that determines the overall health and well-being of a child. The American Academy of Pediatric Dentistry (AAPD) recommends a child’s first dental visit by the age of one or within six months of the first tooth eruption [1]. Adherence to this guideline is vital for establishing a foundation of preventive care, yet awareness and compliance remain low, particularly in middle-income countries. In Saudi Arabia, studies indicate a widespread pattern of dental visits being initiated only in response to problems such as tooth pain or traumatic injury, rather than for preventive purposes [2,3].

Preventive dental visits are essential for early detection of oral health diseases and for reinforcing oral hygiene behaviors such as brushing, dietary management, and application of sealants, and/or fluorides. However, studies from Saudi Arabia reported suboptimal engagement of parents with preventive dental services [4,5,6,7]. Among children in the Eastern Province, 38.3% had never visited a dentist, while a large proportion of patients visited dentists only when experiencing pain [8]. Research from Abha revealed that only 8% of toddlers met the one-year standard guided by AAPD, while most children visited dental clinics between the ages of three and six years [9]. Similarly, Alkhuwaiter et al. (2024) found that over half of the first visits to dentist were seen between the age of three to six years, and the main reason for visit was tooth pain [3].

The benefits of early dental attendance are well-established in literature. Children seen by dentists at the age of 12 months brush more frequently, keep scheduled follow-ups, and maintain better oral hygiene [10]. These visits are linked to fewer cavities and stronger teeth in later childhood. Sabbagh and Alzain (2024) recently found that children seen within their first year had significantly better oral hygiene and a sharp reduction in early childhood caries compared to those whose visits were delayed [11]. These outcomes align with the World Health Organization’s emphasis on timely preventive services to reduce global oral health disparities [12].

Parental understanding and attitudes are essential determinants in oral health of children. A significant knowledge gap exists among Saudi caregivers; for example, in Riyadh, approximately 60% of parents were unaware of the first-year dental visit guideline, often believing a dentist is only necessary for treating caries or pain [2]. Evidence consistently shows that when parents are educated on the timing and purpose of early check-ups, their compliance with professional recommendations improves significantly [13,14]. Furthermore, parental socioeconomic status, particularly education and income, is a strong predictor of oral health behavior. Caregivers with lower income and education levels know fewer preventive tips and delay their child’s first visit by an average of fifteen months [15]. Higher-income households are more likely to attend routine check-ups, confirming economic and educational status as key correlates of preventive health utilization [15,16,17].

Beyond knowledge and socioeconomic factors, practical obstacles such as limited time, travel difficulties, high costs, and a shortage of pediatric dentists regularly delay access [18]. Dental anxiety, affecting both children and parents, further discourages clinic attendance. In the absence of symptoms, parents often perceive dental visits as optional, postponing care until pain arises and shifting the focus from prevention to urgent intervention [19,20,21,22].

A cross-sectional survey revealed that only 43% of parents knew the correct age for a first visit, and while 64% endorsed six-month check-ups, many followed less frequent schedules or were uncertain [2]. Although attitudes toward prevention are often positive, gaps persist in the awareness and practice of measures. School-based interventions, such as providing referral letters after screenings, show promise for improving attendance, though evidence is still limited. Further integration of oral health education into school and primary healthcare systems is a key objective under Saudi Vision 2030’s health transformation.

Nevertheless, despite the recognized burden of oral disease and the advantages of preventive care, a scarcity of data exists quantifying the prevalence of pediatric preventive dental attendance across Saudi Arabia. To tailor effective health promotion policies, it is vital to understand current utilization patterns and identify the sociodemographic and behavioral factors associated with guideline adherence. Therefore, this study aimed to evaluate the timing of the first dental visit among Saudi children and to identify parental barriers and factors associated with compliance with AAPD guidelines.

## 2. Materials and Methods

This cross-sectional study was conducted over three months (14 April to 14 July 2025) at the Pediatric Dental Clinic of the University Dental Hospital, King Saud University, Riyadh, Saudi Arabia. Data was collected using an online, self-administered questionnaire designed to assess parents’ awareness, beliefs, and adherence to the guidelines recommending that a child’s first dental visit should occur by the age of one year, as well as to identify factors influencing adherence.

### 2.1. Setting and Participants

The target sample consisted of parents or guardians of children under 12 years of age. Eligible participants were required to be Saudi citizens and capable of completing a self-administered questionnaire. Exclusion criteria included expatriate parents and unsubmitted or incompletely filled questionnaires. Using convenience sampling, the survey was distributed in pediatric dental clinics and through online platforms. The required sample size was calculated using G*Power version 3.1.9 (Heinrich Heine University, Düsseldorf, Germany) assuming a 95% confidence level, a 5% margin of error, and a prevalence estimate of 50%, which yielded a minimum of 384 respondents [23]. To account for potential non-responses, the sample size was increased by 20%, setting a target of 461 participants. Approximately 520 questionnaires were distributed through both platforms. Finally, 465 complete questionnaires from participants were obtained.

### 2.2. Ethical Considerations

The research protocol was approved by the King Saud University Institutional Review Board (IRB) (Project Number: E-24-9475). Participation in the study was entirely voluntary, and all parents or guardians provided informed consent electronically before completing the questionnaire. No personal identifiers were collected during data collection, and the anonymity and confidentiality of participants were maintained throughout the study.

### 2.3. Questionnaire Development

An Arabic self-administered questionnaire was developed, consisting of four sections and a total of 19 questions. The first page provided information about the study and the questionnaire, along with an informed consent statement for participants. The first section collected demographic information of parents, including age (<25, 25–30, 30–35, 35–40, and >40 years), gender (male, female), education (high school, diploma, bachelor’s, master’s), and monthly income. The second section included five questions assessing parental awareness regarding dental visits and their child’s oral hygiene. The third section comprised six questions related to parental practices and the frequency of dental visits for their children. The fourth section included three questions that explored barriers to dental visits or dental neglect, as well as preferred strategies to improve parental knowledge about the importance of early dental visits.

### 2.4. Translation and Validation

The questionnaire was first translated from English to Arabic, and then backtranslated to English by two certified linguists to ensure accuracy. A pediatric dentist fluent in both languages then compared the original English version with the Arabic translation, making modifications in consultation with the linguists until a finalized Arabic version was established. Test–retest reliability of the Arabic version was assessed through a pilot study involving 20 parents who were not included in the main study. Reliability was evaluated using Pearson’s correlation coefficient, which yielded a value of 0.91, indicating excellent reliability. Internal consistency was measured to assess inter-item correlations, with Cronbach’s α calculated at 0.82. Additionally, four pediatric dentists evaluated the questionnaire items for relevance, clarity, and ambiguity using a four-point Likert scale.

The final version was completed as an online, self-administered Arabic questionnaire via Google Forms and distributed to parents attending pediatric dental clinics at King Saud University. Before participating, all respondents were provided with an online consent form outlining the study’s aims, ensuring that consent was both informed and voluntary.

### 2.5. Data Analysis

Descriptive statistics, including frequencies and percentages, were used to summarize demographic variables. The chi-square test was employed to examine associations between categorical variables, while logistic regression was applied to identify factors associated with compliance with the guideline. Spearman rho correlation coefficients were also calculated to assess relationships among awareness, beliefs, compliance, and perceived barriers. All analyses were performed using SPSS (Statistical Package for the Social Sciences) version 26, with a *p*-value of <0.05 considered statistically significant.

## 3. Results

A total of 465 complete questionnaires were collected out of 520 distributed, resulting in a response rate of 89.8%. Of the respondents, 63.2% were women and 36.8% were men. The majority of participants were aged 30–45 years (37.2%), followed by those aged 25–30 years (29.0%) and those above 45 years (17.7%). In terms of education, the majority held a bachelor’s degree (64.08%), with smaller proportions having completed high school (14.6%), a diploma (14.6%), or postgraduate studies (9.8%). Regarding income, most participants reported earnings between 10,000 and 20,000 SAR (38.7%), while smaller percentages reported lower or higher income levels (Table 1).

**Figure 1:**
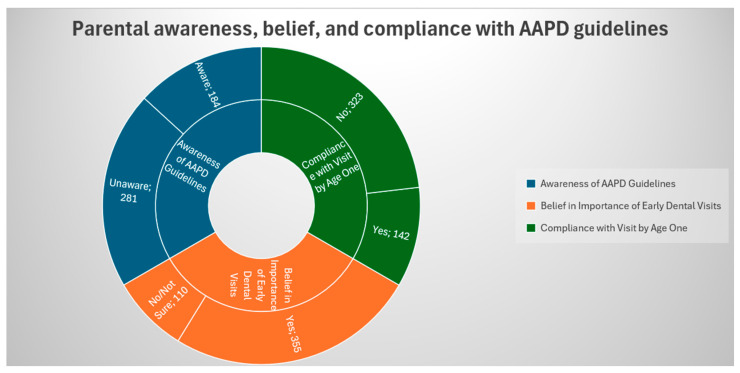
Parental awareness, beliefs, and compliance with AAPD guidelines.

Figure 1 illustrates parental awareness, belief, and compliance with AAPD guidelines. Among 465 respondents, 184 (39.60%) parents were aware of the guidelines, while 281 (60.4%) were unaware. Although 355 (76.30%) parents recognized the importance of early dental visits, only 142 (30.50%) actually complied with the recommendation of taking their child for a dental visit by age one.

**Figure 2:**
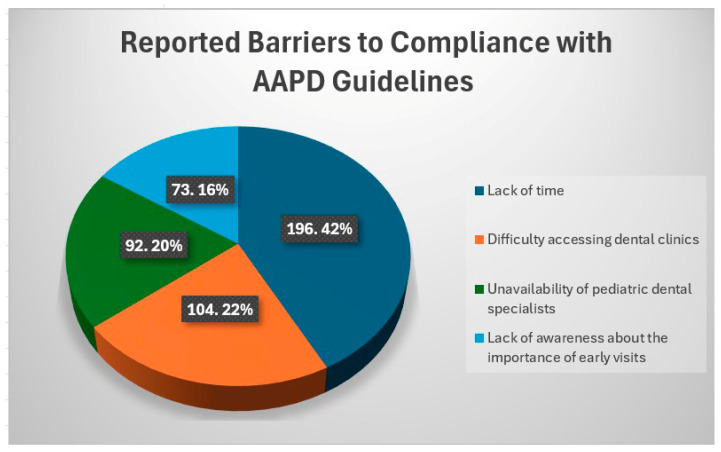
Barriers to compliance with the AAPD guidelines.

The pie chart (Figure 2) illustrates the reported barriers to compliance with AAPD guidelines among participants. The most frequently cited barrier was lack of time (42%, n = 196), followed by difficulty accessing dental clinics (22%, n = 104). Unavailability of pediatric dental specialists was reported by 20% (n = 92) of respondents, while 16% (n = 73) indicated a lack of awareness about the importance of early dental visits. These findings highlight time constraints and access issues as primary obstacles to adherence to pediatric dental care guidelines.

A chi-square test was conducted to determine the difference in the distribution of responses among demographic data (gender, age) and awareness variables (awareness, belief in the importance of early dental visits) with the actual guidelines’ compliance. Based on analysis, parental income (χ^2^ = 20.19, *p* < 0.001) and awareness of AAPD guidelines (χ^2^ = 20.35, *p* < 0.001) were the strongest significant predictors. Education (χ^2^ = 14.25, *p* = 0.003) and Belief in Importance of Early Visits (χ^2^ = 14.53, *p* < 0.001) were also significant, while Age Group was a weaker significant factor (χ^2^ = 12.51, *p* = 0.014). However, gender showed no statistical association (χ^2^ = 0.001, *p* = 0.975) (Table 2).

The socioeconomic variables showed clear associations with adherence to the recommendation of the first dental visit by age one. Both parental education and income had a significant influence on compliance, as higher-educated parents, especially those with postgraduate degrees, exhibited compliance levels of 30.4%. In contrast, diploma holders exhibited the lowest compliance at 1.9%. Furthermore, families earning above 20,000 SAR exhibited the highest compliance rate, at 32.2%, compared to only 7.3% among those with an income of 5000–10,000 SAR. These associations were statistically significant (*p* = 0.003 for education, *p* < 0.001 for income), emphasizing the marked impact of socioeconomic status on caregivers’ engagement in early preventive dental care recommendations (Table 2).

Spearman correlation analysis was conducted to examine the relationships between awareness, beliefs, compliance, and dental visit frequency. A moderate positive correlation was observed between awareness of AAPD guidelines and compliance, although this was not statistically significant (ρ = 0.214, *p* = 0.29). Belief in the importance of early dental visits showed a weak to moderate positive correlation with compliance, which was statistically significant (ρ = 0.183, *p* < 0.02). Additionally, compliance demonstrated a moderate positive correlation with the frequency of annual dental visits, with statistical significance (ρ = 0.318, *p* < 0.06). (Table 3).

**Figure 3:**
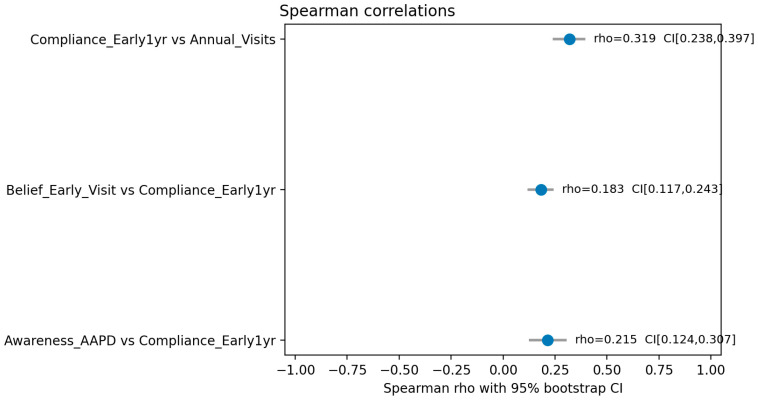
Spearman rho correlation coefficients test the relationship between the categorical and behavioral variables with 95% CI.

In Figure 3, Spearman’s correlation analysis revealed a statistically significant, moderate positive relationship between compliance with the early one-year dental visit recommendation and the frequency of annual visits (ρ = 0.319, 95% CI [0.238, 0.397]). Weaker, yet significant, positive correlations were also observed between compliance and both awareness of AAPD guidelines (ρ = 0.215) and belief in the importance of early visits (ρ = 0.183).

## 4. Discussion

The main role of the pediatric dentist is to prevent, protect, and treat the oral and dental health of a child from the prenatal period up to 15–16 years of age. However, maintaining a child’s oral health does not depend solely on pediatric dentists; it also requires consistent parental involvement. Parents play a crucial role in preventing oral diseases, ensuring adherence to preventive measures, and following the guidelines provided by organizations such as the WHO and AAPD [1,12]. Parental attitudes and knowledge about dental care and early dental visits are therefore key factors directly influencing children’s oral health [22]. The current study aimed to evaluate the timing of the first dental visit among Saudi children and to identify parental barriers and factors associated with compliance with AAPD guidelines.

Since parents are most important social force affecting child’s development during early childhood period, the interventions aiming to improve parental knowledge and awareness are proven to be effective in preventing child’s oral health. One of the major barriers reported in the study was lack of awareness amongst the parents. Around 16% of parents in the included study were not aware of AAPD guidelines for early visit to dental clinics. Similar findings were reported in the study by Karagöz Doğan et al., in which parents failed to visit dental clinics during early age of child due to lack of awareness [24]. This can be attributed to lack of information provided to the parents by healthcare and social workers on the importance of oral health.

One of the interesting findings of the current study was the awareness of the guidelines provided by AAPD regarding first dental visit of a child during the age of 6 months to one year. In total, 39.60% of parents in the study were aware of these guidelines, but only few of them have followed the guidelines. In a study conducted in Turkey, only 22.2% of parents were aware of these guidelines. In the current study, 60.6% of parents reported visiting dentists only when complaints arise [24]. Similarly, in a study by Tokuç et al., it was reported that the first dental visit for maximum children occurred between 4 and 6 years, and that was due to pain or traumatic injury [25]. In a study by Alaa et al., it was reported that most parents believe that the first dental visit of a child should be at the age of 3 or 4 years, not before that [10]. However, in this study, 184 included parents believed that the first dental visit of their child must be before the age of one year. These findings indicate that most of the parents in the Riyadh region are unaware of early dental visits and believe that the first dental visit of a child should be at preschool age. Hence, there is the requirement for parental educational programs to ensure that preventive oral health measures are provided to each child.

The study also demonstrated a significant association (*p* = 0.001) between parental education, income, and awareness and compliance with preventive oral healthcare. Similar findings were reported by Hussein et al., in which higher parental education was associated with greater awareness of preventive practices [10]. Previous studies confirm that lower educational levels contribute to delays in seeking dental care for children [14,26,27,28]. Additionally, families with lower socioeconomic status often face financial barriers that deprioritize children’s oral health needs [12]. Although a substantial proportion of parents in this study agreed that the first dental visit should occur between 6 months and 1 year, their responses to the question, “When did your child first visit the dentist?” revealed inconsistencies, with many delaying the first visit. Comparable inconsistencies were also noted in a cross-sectional study conducted in Turkey [25].

A positive finding of this study highlights the correlation between early dental visits and compliance to follow AAPD guidelines. This finding suggests that parents of toddlers are ready to follow the guidelines and are willing to bring their children for dental checkups on regular basis. This may be the sign that parents are well aware of the importance of early checkups before any dental problem arises. Similar findings were reported in a questionnaire study from Belagavi, India [28]. In this study, a significant number of parents agreed to follow-up visits to dentists. However, 30.3% of parents were not interested in annual follow up; this could be attributed to the underestimation of the value of follow-up visits or sparing little time to take additional visits for their children [28]. This is similar to the finding in the current study, in which around 76% of parents identified lack of time as barrier to dental visits. Studies have reported that timely and early dental visits improve oral health outcomes [16,17,18]. This point emphasizes that dentists and other healthcare professionals should provide accurate knowledge to the parents regarding the importance of early dental visits.

The findings of this study emphasize the importance of increasing parental awareness of AAPD guidelines and promoting early and regular dental checkups. Community-based educational programs should be strengthened to encourage preventive behaviors and reduce barriers to care. Furthermore, health policies should ensure accessible and cost-effective pediatric dental services, thereby enabling parents to adopt preventive measures that can significantly improve children’s oral health.

### 4.1. Strengths and Implications

This is the first study in Saudi Arabia to evaluate parental knowledge regarding the first dental visit of their child, highlighting the importance of preventive care among parents. By considering parental education and family income, the study further analyzes parental attitudes toward early dental visits. This represents the strength of the research, as it assesses the impact of socioeconomic status on children’s oral health. These strengths enhance both the scientific validity and societal relevance of the study. The findings also indicate the need to develop awareness programs for parents and improve their access to pediatric dental clinics. Future research should focus on comprehensive strategies to improve parental knowledge of early dental visits and preventive measures. Such strategies could include digital educational materials for pregnant mothers, one-on-one counseling sessions, and school-based dental programs. These initiatives would ultimately increase parental awareness and improve children’s oral health outcomes.

### 4.2. Limitations

Despite its strengths, the study has certain limitations. Firstly, the use of an online survey may have introduced selection bias. Although forms were distributed to parents of toddlers visiting dental clinics at King Saud University, the number was small compared with online distribution. Online dissemination likely excluded parents from rural areas with limited internet access, thereby reducing the generalizability of the findings. A larger, more representative sample size would have provided stronger conclusions regarding the barriers to early dental visits. Another limitation is reliance on self-reported data without verification through children’s clinical records, which may have resulted in over- or underestimation of parental awareness. Finally, the study did not consider the number or ages of children in each family, which could influence parental awareness since parents with older children may already be more familiar with the importance of preventive dental care.

## 5. Conclusions

The findings of the current study revealed that while many parents acknowledged the importance of early preventive care, actual compliance with the recommended first dental visit by age one remained low (30%). Lack of awareness, limited time, and access constraints were identified as the major barriers, while higher levels of parental education and income were significantly associated with greater adherence to preventive oral healthcare.

These results underscore the critical role of parental awareness and socioeconomic status in shaping children’s oral health behaviors. Targeted educational initiatives, such as digital resources for new and expecting parents, school-based interventions, and counseling by healthcare professionals, are needed to bridge the gap between awareness and practice. At the policy level, expanding access to affordable pediatric dental services and integrating preventive oral health programs within primary healthcare frameworks are essential steps to encourage timely dental visits.

## Figures and Tables

**Table 1:** Demographic distribution of participating parents (n = 465).

Variable	Category	Frequency (*n*)	Percentage (%)
Gender	Women	294	63.20%
	Men	171	36.80%
Age Group	Under 25 years	75	16.10%
	25–30 years	135	29.00%
	30–45 years	173	37.20%
	Over 45 years	82	17.70%
Education	High school	68	14.6%
	Diploma	68	14.6%
	Bachelor	298	64.08%
	Postgraduation	46	9.8%
Income	Less than 5000	102	21.9%
	5000–10,000	124	26.66%
	10,000–20,000	180	38.7%
	More than 20,000	59	12.6%

**Table 2:** Chi-square test for association between demographic and awareness variables with compliance.

Variable	χ^2^ Value	*p*-Value	Statistical Significance
Gender	0.001	0.975	Not Significant
Age Group	12.51	0.014 *	Significant
Education	14.25	0.003 *	Significant
Income	20.19	<0.001 **	Highly Significant
Awareness of AAPD Guidelines	20.35	<0.001 **	Highly Significant
Belief in Importance of Early Visits	14.53	<0.001 **	Significant

** *p*-value highly Significant; * *p*-value significant.

**Table 3:** Spearman rho correlation coefficients test the relationship between the categorical and behavioral variables.

Variable Pair	Spearman Rho	*p*-Value	Interpretation
Awareness of AAPD Guidelines vs. Compliance	0.215	0.29	Moderate positive correlation
Belief in the Importance of Early Visit vs. Compliance	0.183	<0.02	Weak to moderate positive correlation
Compliance vs. Frequency of Annual Dental Visits	0.318	<0.06	Moderate positive correlation

## Data Availability

The data presented in this study are available on request from the corresponding author. The data are not publicly available due to institutional and ethical restrictions regarding participant confidentiality.
